# Supplementary material for: Hybrid Models and Biological Model Reduction with PyDSTool
Source: PLoS Comput Biol. 2012 Aug 9;8(8):e1002628. doi: 10.1371/journal.pcbi.1002628 (PMC3415397; doi:10.1371/journal.pcbi.1002628)
Supplement: Text S4 — Complete source code for the PyDSTool package (version 0.88.120504). Includes API documentation and help files linking to web pages. This file is identical to the current public release on Sourceforge.net. (ZIP) [file pcbi.1002628.s004.zip › PyDSTool/html/PyDSTool.Toolbox.optimizers.criterion-pysrc.html]

xml version="1.0" encoding="ascii"?


PyDSTool.Toolbox.optimizers.criterion


| Home | Trees | Indices | Help | PyDSTool |
| --- | --- | --- | --- | --- |

Package PyDSTool ::
Package Toolbox ::
Package optimizers ::
Package criterion

[hide private]

[frames] | no frames]

# Source Code for Package PyDSTool.Toolbox.optimizers.criterion

```
 1   
 2  # Matthieu Brucher 
 3  # Last Change : 2007-08-24 10:04 
 4   
 5  """ 
 6  Module containing every criteria for converge test 
 7   
 8  Functions : 
 9    - crietrion() creates a composite criterion 
10   
11  Criteria : 
12    - IterationCriterion 
13      - stops when the iteration limit is reached 
14    - MonotonyCriterion 
15      - stops when the cost rises again 
16    - RelativeValueCriterion 
17      - stops when the relative value error is below a certain level 
18    - AbsoluteValueCriterion 
19      - stops when the absolute value error is below a certain level 
20    - RelativeParametersCriterion 
21      - stops when the relative parameters error is below a certain level 
22    - AbsoluteParametersCriterion 
23      - stops when the absolute parameters error is below a certain level 
24    - GradientCriterion 
25      - stops when the gradient is below a certain level 
26   
27  Composite criteria : 
28    - OrComposition 
29      - returns True if one of the criteria returns True 
30    - AndComposition 
31      - returns True if all the criteria return True 
32   
33  Information criteria : 
34    - AICCriterion 
35      - stops when the cost function increases again 
36      - is dependent of the number of parameters if it changes 
37    - ModifiedAICCriterion 
38      - Identical to AICCriterion except that a number of increases are tolerated 
39  """ 
40   
41  from criteria import * 
42  from composite_criteria import * 
43  from information_criteria import * 
44  from facilities import * 
45   
46  criterion__all__ = ['IterationCriterion', 'MonotonyCriterion', 'RelativeValueCriterion', 'AbsoluteValueCriterion', 'RelativeParametersCriterion', 'AbsoluteParametersCriterion', 'GradientCriterion', 
47                      'OrComposition', 'AndComposition', 
48                      'AICCriterion', 'ModifiedAICCriterion', 
49                      'criterion', ] 
50   
51  __all__ = criterion__all__ 
52
```

  


| Home | Trees | Indices | Help | PyDSTool |
| --- | --- | --- | --- | --- |

|  |  |
| --- | --- |
| Generated by Epydoc 3.0.1 on Fri May 4 15:24:24 2012 | http://epydoc.sourceforge.net |
